# Prioritizing Targets and Minimizing Distraction Within Limited Capacity Working Memory: Commentary on ‘Working memory and attention; a conceptual analysis and review’ by Klaus Oberauer

**DOI:** 10.5334/joc.75

**Published:** 2019-08-08

**Authors:** Richard J. Allen

**Affiliations:** 1 School of Psychology, University of Leeds, Leeds, UK

**Keywords:** Working memory, Attention, Short-term memory

## Abstract

Oberauer (2019) maps out different perspectives that have emerged in exploring working memory and attention, and suggests particular ways in which these key aspects of cognition might operate in the service of successful goal completion. One question that is central to Oberauer’s review and to the field more generally concerns how automatic and controlled attention interact with each other and with working memory. In line with this, recent research indicates that both forms of attention can operate within the same task to determine whether information is maintained in working memory. Perceptual attention can be automatically captured by environmental input, resulting in superior recall for the most recent stimulus, along with unwanted disruption by distracting stimuli. Effortful top-down control, powered by executive resources, operates within this context to create and maintain task goals, and to support the maintenance of target information in an accessible state, particularly if it is of greater value/goal relevance.

The relationship between working memory and attention has been the focus of a wealth of research over recent years, in efforts to understand how these core components of cognitive function might operate and whether they might be conceptualized as part of the same broad system responsible for driving perception, thought, and action. Oberauer (2019) sets out a timely review of relevant literature and a new taxonomy, with a view to better describing and understanding this relationship. The starting point lies at a possible distinction between two broad approaches, namely, attention as a limited resource and as selective information processing. This is indeed a useful distinction to draw in helping characterize different perspectives within the literature, though each of these viewpoints are clearly likely to have value. Attention is a limited resource, placing constraints on how many ‘non-perceptual’ items can be maintained, and thus contributing to the limited capacity typically seen as a hallmark of working memory. This limited capacity then underlines the importance of appropriate ‘perceptual’ selection to meet task goals and avoid non-perceptual, mnemonic representations being overloaded and displaced. Indeed, bottlenecks at encoding and consolidation are a useful constraining factor in controlling undesired representational turnover. In this way, limits on both control and selection might work reciprocally. The end result is an efficient system that makes the most of its’ constraints, optimizing performance by retaining recent and salient information (automatically derived from the environment) and/or information aligned to current goals (selected through controlled processing). It remains to be seen whether limits operate on the control of processes, as suggested by Oberauer, or on the processes themselves, though empirically distinguishing between these possibilities may prove challenging.

Evidence from dual-task methodology indicates that a domain-general, executive-related form of attention contributes to working memory performance (e.g. Allen, Baddeley, & Hitch, 2006, 2014; Morey & Bieler, 2013). Oberauer (2019) acknowledges this role, though omits executive attention from his taxonomy, noting the ambiguity of the term, and how it relates both to the processing of goals, action planning, and response selection, and also to top-down control of attention towards information in the environment. Of course, these dimensions are likely to be related; top-down control is typically driven by our goals and how we implement plans and responses in order to meet those goals, and each draw on a common, limited executive-based attentional resource. Given the clear evidence supporting the contribution of top-down, effortful control to visual working memory, a key question then is how this might be involved in directing attention away from distraction, and towards targets, within working memory tasks. Our work has demonstrated that the presence of to-be-ignored (TBI) items captures ‘perceptual’ attention and negatively impacts on working memory (Allen, Baddeley, & Hitch, 2017; Ueno, Allen, Baddeley, Hitch, & Saito, 2011), with such interference possibly disrupting items within the focus of attention (Hu, Hitch, Baddeley, Zhang, & Allen, 2014). However, this disruption does not seem to be controlled in a top-down, effortful, cognitively demanding manner. Interference arising from TBI stimuli encountered either concurrently with targets (Allen et al., 2017) or after target offset (Hu, Allen, Baddeley, & Hitch, 2016) appears to be broadly unchanged when dual-task load is increased.

Any new, salient, perceptual information may have a certain probability of being automatically encoded, especially if it contains features that are potentially goal-relevant (Ueno et al., 2011). If such a stimulus represents a target item, it will be recalled more accurately if it is the final item in a sequence (Allen et al., 2006). If it is a TBI distractor, it will normally be filtered out, though with less than perfect efficacy. Following the possibly effortful process of creating a task set that determines goals and criteria for target feature detection, filtering out perceptual distraction appears to operate without further loading on control processes. Thus, in accord with Oberauer (2019), control of perceptual distraction may be distinct from other forms of attentional control involved in working memory.

Instead, controlled attention may be particularly important in encoding and maintaining goal-relevant targets. Following Oberauer, this may be limited by the extent to which control can be exerted across targets, particularly when memory load and/or concurrent processing demands are high. In these contexts, focusing limited resources on subsets of items is useful (Atkinson, Baddeley, & Allen, 2018), in order to optimise the efficient use of limited resources, particularly when items vary in their goal relevance or associated value. There are likely to be multiple ways in which attention can be directed within a working memory task (Atkinson et al., 2018). Our own work on directed prioritization has shown that, when certain targets are associated with increased points values, participants can direct attention to those targets to enhance their recall probability, possibly retaining them within the focus of attention (e.g. Hu et al., 2014, 2016). It also appears that participants can prioritise more than one item at a time (Allen & Ueno, 2018; Hitch, Hu, Allen, & Baddeley, 2018), suggesting a multiple-item focus of attention, or at least the ability to rapidly circulate items through such a focus to keep them active and accessible. While further work is necessary, such an ability might draw on effortful top-down control (Hu et al., 2016). It also appears that those items active within focused attention are more vulnerable to interference from automatically derived, perceptual input (Allen & Ueno, 2018; Hu et al., 2014, Hitch et al., 2018). Thus, the contents of focused attention reflect tension between controlled and automatic, perceptual and non-perceptual information.

Prioritization involves controlled direction of selective attention. Drawing on Oberauer’s taxonomy, it involves controlled direction of perceptual attention to items in the environment, though it remains to be seen how encoding of high- and low-priority items might differ, and whether this simply reflects variation in the probability with which each type of item is both spatially attended and actively processed. As Oberauer notes, spatially attending to an item does not mandate working memory storage. High priority items may be more likely to be actively encoded. Non-perceptual attention would then be directed to these items when held in working memory, possibly as a form of visualisation or attentional refreshing through the focus of attention. Attentional control will also be important in maintaining task set, ongoing monitoring of goals and goal-progress, and preparing and implementing response selection. With attentional focus directed elsewhere (e.g. to non-perceptual processing), the probability of creating and maintaining a robust representation of low value items would be reduced, even if they are initially spatially attended.

Overall, Oberauer’s review provides a useful framework in which to consider how different perspectives on working memory and attention might be integrated, and how these processes operate to support successful action. In aiming to develop a more nuanced and precise view of what can be derived from the expanding literature, this review should help guide future exploration, though it remains possible that certain distinctions between perspectives or processes might turn out to represent different angles on the same basic question. Work by our group on this topic speaks directly to Oberaeur’s conceptual analysis; both automatic and controlled attention contribute to determine which items are encoded and maintained in working memory, with the push and pull between these functions serving to increase the probability of successful goal completion within the constraints of these limited capacity systems.
